# Online Dissemination Strategies of a Canada Research Chair: Overview and Lessons Learned

**DOI:** 10.2196/resprot.6413

**Published:** 2017-02-24

**Authors:** Jessica Hébert, Hubert Robitaille, Stéphane Turcotte, France Légaré

**Affiliations:** ^1^ Centre Hospitalier de l'Université de Québec Research Centre Population Health and Practice-Changing Research Group Quebec City, QC Canada; ^2^ Centre Hospitalier de l'Université de Québec Research Centre Department of Family Medicine and Emergency Medicine Université Laval Quebec city, QC Canada; ^3^ Centre Hospitalier de l'Université de Québec Research Centre Population Health and Practice-Changing Research Group Quebec city, QC Canada

**Keywords:** social media, knowledge translation, shared decision making, online dissemination, knowledge dissemination

## Abstract

**Background:**

Little is known about the use of online dissemination strategies, such as websites and social media, to increase the visibility and uptake of research.

**Objective:**

To describe two online dissemination strategies of the Canada Research Chair in Implementation of Shared Decision Making in Primary Care over an eight-year period.

**Methods:**

Our two sources of online dissemination data were the website of the Canada Research Chair in Implementation of Shared Decision Making in Primary Care and the Chair’s Twitter account. We conducted a content analysis of the news section of the website. We extracted website usage statistics using Google Analytics and analyzed indicators such as total number of visits, new and returning visitors, page views per visit, time spent onsite per visit, visitors’ country of origin, and most popular pages. From the Chair’s Twitter account, we collected the number of tweets, followers, and follows. We consulted Google Scholar to chart the trend in citations of the Chair’s articles over the same period.

**Results:**

From the website’s inception in January 2008 to December 2015, we recorded an average of 7906 visits per year (3809 in 2008; 8874 in 2015), 65.85% of which involved new visitors (5206/7906). The average number of pages viewed per visit was 3.2 and average bounce rate was 57.87% (4575/7906). Visitors spent an average of two minutes and 12 seconds per visit. We computed visits from 162 countries, with the majority from Canada (5910/7906, 74.75%). In order of frequency, the seven most visited pages were: (1) home page with news of publications and grants (24,787 visits), (2) profile of Chairholder (8041 visits), (3) profiles of research team members (6272 visits), (4) list of research team members (4593 visits), (5) inventory of shared decision making (SDM) programs (1856 visits), (6) interprofessional approaches to SDM (1689 visits), and (7) description of Chair activities (1350 visits). From the inception of the Twitter account in April 2011 to November 30, 2016 we recorded 5831 tweets in French and English, 1679 followers, and 1112 follows. The total number of visits and visitors to the website increased during the first three years, stabilized, and then dropped slightly, while the number of returning visitors rose slightly. In comparison, citations of the Chair’s articles increased steadily over the same period, rising more sharply as visits to the website declined.

**Conclusions:**

Over an eight-year period, visitors to the website increased in the first three years before levelling off. Meanwhile, the Chair’s citations rose continuously. There was no observable association between website visits or Twitter activity and rising citations. Our results suggest that online dissemination may not yet be a major determinant of research uptake or visibility in the scientific community.

## Introduction

Knowledge dissemination strategies are essential for moving health services research into practice [1-4]. Traditional strategies include peer reviewed publications and conferences. Newer online methods [5,6] include passive platforms (eg, websites) as well as more active social media platforms (eg, Twitter) that allow the creation and exchange of user-generated content [7]. While research teams are increasing their use of online dissemination strategies, little is known about their influence on the visibility and uptake of research results [8-12].

The Canada Research Chair in Implementation of Shared Decision Making in Primary Care, based in Quebec City, was created in June 2006 (evolving into the Canada Research Chair in Shared Decision Making and Knowledge Translation in 2016) [13]. The mission of the Chair is to provide health professionals and their patients with the necessary skills to engage in shared decision making (SDM) throughout the healthcare continuum, and it aspires to be a world-class training and support center for the implementation of SDM in Canada and abroad. In March 2008, the Chair began to invest in online dissemination in both French and English, and has been doing so ever since. We sought to describe the use of two of the Chair’s online dissemination strategies over an eight-year period.

## Methods

### Sources of Data

Our three sources of retrospective data were: (1) the website of the Chair [14] and site statistics obtained from Google Analytics; (2) the Chair’s Twitter account (SDM_ULAVAL); and, for comparison purposes, (3) Google Scholar citations data on the Chair’s articles [15]. The website was created in March 2008 at Laval University and is managed by a French speaking webmaster who spends approximately four hours per week populating, translating, and updating it. English pages are reviewed by an English editor. The Twitter account was created in 2011 by the Chairholder, who is solely responsible for its content.

### Data Collection

From the website’s inception in March 2008 to December 31, 2015, we collected data on the news section and classified it into five categories: (1) publications; (2) honors, awards, and scholarships; (3) grants; (4) congress and conference announcements; and (5) others. We extracted usage statistics from Google Analytics from January 1, 2008 to December 31, 2015, including: (1) number of visits, (2) number of visitors, (3) new visitors, (4) returning visitors, (5) page views per visit, (6) bounce rate, (7) time spent on the site per visit, (8) country of origin of visitors, and (9) most popular pages. The bounce rate represents the percentage of visitors who enter the site and then leave, rather than continuing to view other pages. We collected data from the Chair’s Twitter account on November 30, 2016 on number of tweets, number of followers, and follows since its inception in April 2011. We collected the average number of tweets per month over 21 months using TweetStats [16]. We also collected data from Google Scholar on citations of the Chair’s research articles from March 2008 to December 2015.

### Data Analysis

We performed content analysis of the website based on the five categories of the news section. Descriptive statistics such as frequency distributions, means, and standard deviations were calculated to summarize indicators of the website usage from Google Analytics. We charted citations of the Chair’s articles over the same period.

## Results

### Website

**Figure 1:**
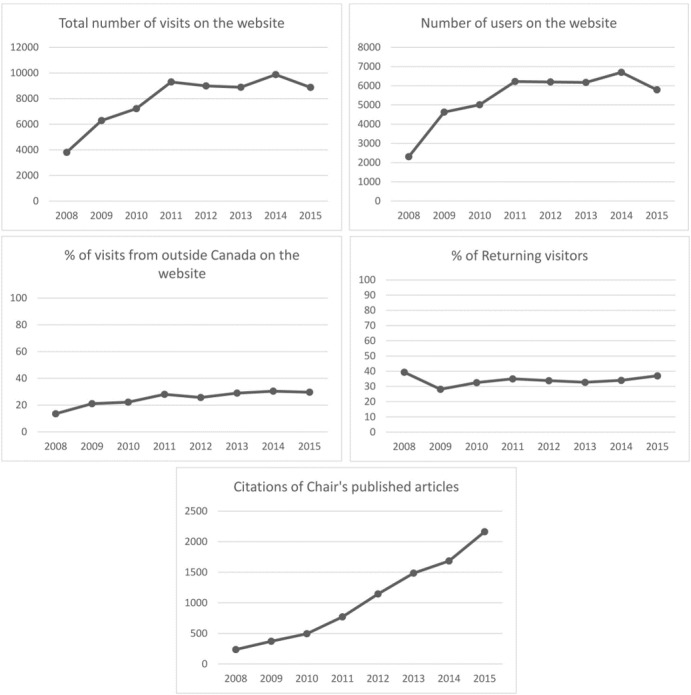
Graphic representation of indicators of the website usage from Google Analytics and citation of Chair’s published articles over eight years.

As of December 31, 2015, we recorded 184 news items: 79 regarding publications; 35 regarding honors, awards, and scholarships; 22 regarding grants; 45 regarding conference announcements, and 3 regarding various other topics. We recorded an average of 7906 visits and 5382 users per year (See Table 1 and Figure 1), and 65.85% (5206/7906) of visits were from new visitors. The average number of pages viewed per visit was 3.2, and the average bounce rate was 57.87% (4575/7906). Visitors spent an average of 2 minutes and 12 seconds per visit. The number of visits and visitors increased during the first three years (from 3809 visits and 2311 visitors in 2008 to 9301 visits and 6224 visitors in 2011), remained stable from 2011 to 2014, and then dropped slightly to 8874 visits and 5789 visitors in 2015. We computed visits from 162 countries over the eight years, with the majority from Canada (5910/7906, 74.75%). The seven most visited pages were: (1) home page with news of new publications, awards, and grants, as well as the Chair’s Twitter feed (24,787 visits); (2) profile of the Chairholder (8041 visits); (3) profiles of team members (6272 visits); (4) list of team members (4593 visits); (5) inventory of SDM programs for healthcare professionals (1856 visits); (6) interprofessional approaches to SDM (1689 visits); and (7) description of Chair activities (1350 visits). All other pages were consulted fewer than 1000 times.

**Table 1:** Indicators of the website usage from Google Analytics and citation of Chair’s published articles

Year	Total number of visits (n)	Number of users (n)	New visitors (%)	Returning visitors (%)	Page views per visit (n)	Bounce rate (%)	Time spent on site per visit (minutes)	Visits from outside Canada (%)	Citations of Chair’s published articles
2008	3809	2311	60.6	39.4	4.70	40.12	3:19	13.60	238
2009	6289	4634	71.8	28.2	3.42	56.32	2:18	21.18	372
2010	7215	5014	67.4	32.6	3.14	58.28	2:01	22.27	495
2011	9301	6224	64.9	35.1	3.07	61.35	2:04	28.09	773
2012	8992	6203	66.1	33.9	2.90	63.21	1:43	25.78	1147
2013	8891	6178	67.2	32.8	2.69	60.72	1:55	28.96	1486
2014	9877	6702	65.9	34.1	3.09	61.88	2:45	30.45	1685
2015	8874	5789	62.9	37.1	2.85	61.07	2:15	29.70	2164
Average over 8 years	7906	5382	65.9	34.2	3.23	57.87	2:12	25.00	1045

We observed an increase in the total number of visits and visitors to the website in the first three years, then a stabilization until 2015 when the numbers dropped slightly. In the final year, the percentage of returning visitors increased slightly, but this finding was not statistically significant. There was a slight downward trend for visits to the home page, with an increasing bounce rate.

### Citations

Citations of the Chairholder’s articles increased from 238 to 2164 (800%) over the eight years.

### Twitter

We recorded an average of 100 tweets per month on SDM_ULAVAL, the Chair’s Twitter account, from April 2014 (as far back as the tool permitted) to December 2015. As of November 30, 2016, the account had 5831 tweets, 1679 followers, and 1112 follows.

## Discussion

This study retrospectively describes the implementation of two online dissemination strategies of a Canada Research Chair, namely a website and a Twitter account. We observed that over an eight-year period visitors to the website increased most in the first three years, levelled off, and then dropped slightly in the final year. The percentage of visitors from outside Canada dropped less in the final year, and the percentage of returning visitors rose slightly. Meanwhile, citations of the Chair’s research articles rose steadily.

The early rise in visits to the Chair’s website followed by stabilization may reflect the fact that online strategies are ephemeral tools that may have immediate, rather than lasting, impact [14]. The high bounce rate and recent downward trend in visits to the website may reflect the increasingly stiff competition in research visibility. It appears that for these knowledge translation tools to be effective, they require an increasingly aggressive and time-consuming online presence. Conversely, webmetrics cannot compute time spent on bounced pages, and as the home page contains regularly updated news about publications and a live feed of the Chair’s Twitter account, visitors may have felt that they needed to look no further. The slight rise in percentage of returning visitors and the high number of Twitter followers suggest that the Chair’s online dissemination strategies may have attracted a faithful following.

Our results showed no direct correlation between the Chair’s citations and usage of the website and Twitter account. While some authors have shown significant correlation between social media mentions and download and citation counts [14,15], they also note the difficulty of collecting appropriate data, and warn that citation levels of manuscripts are just as likely to reflect their scientific quality or popular appeal. Many scientists consider the value of Twitter to be, first, “a constant live literature alert service crowdsourced from peers,” and second, its social impact, which complement (rather than increase) citations [15]. Our results show that our website may perform a similar complementary function: visitors’ second main interest after the home page showing news of the Chair (24,787 visits) was in lists and profiles of team members (18,906 visits). This finding suggests that an online presence may be as important for scientific networking as it is for direct uptake of results.

### Limitations

The Chair website was not launched until March 2008, meaning that data for the complete year was missing and total traffic was therefore underrepresented. In addition, we did not chart the evolution of Twitter data for the entire study period.

### Conclusions

We could not determine a direct link between our online dissemination strategies with increased research uptake or visibility over time. This finding suggests that online dissemination may not yet be a major determinant of research uptake or visibility in the scientific community. However, appropriate metrics (including measures of social impact) and high-quality data are needed to understand the full impact of these tools and discover the most effective ways to use the various platforms.

## Abbreviations

SDM: shared decision making
